# Reducing the Number of Distractors in Multiple-Choice Questions: A Randomized Study in Undergraduate Medical Education

**DOI:** 10.5334/pme.2583

**Published:** 2026-04-22

**Authors:** Patricia Sunsundegui, Marcos Llorente-Ortega, Felipe Lucena, Nerea Fernández-Ros, Maite Solas, Ana Belén Alcaide, Manuel F. Landecho, Jorge Quiroga, Mercedes Iñarrairaegui, José Ignacio Herrero

**Affiliations:** 1 Department of Internal Medicine, Clínica Universidad de Navarra, Av. Pio XII, 36, 31008 Pamplona, Spain; 2 Department of Science, Nuestra Señora del Pilar School, 42001 Soria, Spain; 3 Department of Pharmaceutical Sciences, University of Navarra, Pamplona, Spain; 4 Instituto de Investigación Sanitaria de Navarra (IdiSNA), Pamplona, Spain; 5 Department of Respiratory Medicine, Clínica Universidad de Navarra, Pamplona, Spain; 6 Liver Unit, Clínica Universidad de Navarra, Pamplona, Spain; 7 Centro de Investigación Biomédica en Red de Enfermedades Hepáticas y Digestivas (CIBERehd), Pamplona, Navarra, Spain

## Abstract

**Background:**

Multiple-choice questions (MCQs) are widely used in medical education due to their efficiency and scalability. However, the quality of distractors plays a critical role in item validity. Evidence suggests that reducing distractors may enhance psychometric performance without compromising assessment quality.

**Methods:**

We conducted a randomized study involving 198 MCQs used in second-year medical exams at the University of Navarra. Each exam included 50 items with two distractors and 50 with three distractors. Items were analyzed for difficulty index, point-biserial coefficient, low-functioning distractors, item-writing flaws, and overall quality (difficulty index 0.45–0.75 and point-biserial > 0.20). In addition, items were classified as either factual recall or clinical case-based to assess the effect of question type. Univariate and multivariate analyses were performed.

**Results:**

No significant differences were found between two- and three-distractor items in terms of difficulty, discrimination, or overall quality. However, items with two distractors presented fewer item-writing flaws (60.2% versus 46%; p = 0.045) and more frequently all their distractors were functional (38.8% versus 18%; p = 0.01). Factual questions were easier and more likely to contain poor distractors, while clinical-case items were more complex and discriminative. Multivariate analyses confirmed that distractor quality, not quantity, was the main factor associated with item performance.

**Conclusions:**

Multiple-choice questions with two well-constructed distractors are a valid and efficient alternative to traditional formats. Reducing the number of distractors improves distractor quality and reduces writing flaws, without compromising psychometric standards. These findings support a shift toward more streamlined item formats in medical education assessment.

## Introduction

The assessment of theoretical knowledge in medical education has traditionally relied on multiple-choice questions (MCQs) due to their efficiency in covering broad curricular content and their simplicity of grading in large student cohorts. However, numerous studies have highlighted quality deficiencies in many MCQs, potentially compromising their ability to discriminate between students with different levels of knowledge [1,2]. A commonly reported flaw is the presence of non-functioning or low-functioning distractors—those that are never or rarely selected by students—which undermines the discriminatory validity of the item [1].

Several studies have questioned the value of including a large number of distractors in MCQs. A systematic review by Rodríguez et al. [3] demonstrated that three-option questions (one correct answer and two distractors) are comparable, and in some cases superior, to four- or five-option formats in terms of validity and reliability. Other studies have found that reducing the number of distractors is associated with higher distractor quality and a lower incidence of item-writing flaws [4,5]. This finding is particularly relevant from a practical standpoint, as generating plausible and functional distractors remains one of the most challenging aspects of MCQ construction [6].

A recent study conducted by our group [7] analyzed 257 four-option MCQs used in pathophysiology exams in an undergraduate medical program. Over 75% of the items included at least one low-functioning distractor, and questions based on clinical cases exhibited greater discriminatory power than factual questions. This finding is consistent with previous evidence [8] and aligns with pedagogical principles focused on clinical reasoning and problem-solving.

Building on this evidence, recent years have also seen significant curricular reforms in medical education aimed at promoting contextualized, progressive learning centered on the acquisition of clinical and transversal competencies. These reforms advocate for the integration of content across disciplines and the alignment of basic and clinical sciences to facilitate deep and lasting learning. Furthermore, they emphasize student-centered approaches, incorporating active learning strategies such as problem-based learning, collaborative learning, and flipped classrooms. A cornerstone of these new educational models is the need to ensure that assessment systems accurately and fairly reflect students’ levels of competency. This imperative requires a critical reassessment of traditional assessment methodologies and underscores the need to examine whether these approaches remain fit for purpose, or if targeted modifications could enhance both item discrimination and student learning.

Recent evidence from a systematic review and network meta-analysis suggests that three-option MCQs demonstrate comparable psychometric performance to four- or five-option formats, while offering greater efficiency and reduced risk of non-functioning distractors [15]. However, the certainty of evidence was rated as low, and heterogeneity across educational contexts remains substantial. These findings underscore the need for well-designed randomized studies conducted within authentic educational settings.

In light of this need to improve the quality and effectiveness of assessment strategies, and building on previous evidence, the primary objective of our study was to evaluate whether MCQs with two distractors demonstrated superior discriminatory capacity compared to those with three distractors, using a randomized design. Additionally, we assessed the impact of question type (factual versus clinical case-based) and the number of low-functioning distractors on item quality.

## Methods

### Context

Since the 2020/2021 academic year, the undergraduate medical program at the University of Navarra is structured to include an initial preclinical cycle (Years 1 and 2), which focuses on the basic foundations of human morphology, structure, and function in health and disease, as well as research training; a clerkship-based third year; and a clinical cycle encompassing years 4 through 6. System and Organ Pathology I and II are taught in the second year under the coordination of the Department of Internal Medicine. They are two-month courses integrating content from pathophysiology, radiology, pathology, and pharmacology. System and Organ Pathology I covers acid-base balance, the renal and urinary systems, cardiovascular system, and hematology, while System and Organ Pathology II includes the respiratory and digestive systems, hepatology, endocrine and metabolic diseases, and musculoskeletal and dermatological pathology. Both courses conclude with a two-part final written examination: a 100-item multiple-choice section and a section comprising five narrative questions. Each section contributes equally to the final course grade.

### Study Design

A randomized study was conducted during the 2022–2023 academic year to compare the discriminative capacity of multiple-choice questions with two versus three distractors in the exams of both courses in the second year of the medical degree program.

All questions were initially written with four response options (one correct answer and three distractors). Half of the questions on each exam were randomly selected, and the author of each item was asked to remove the least relevant distractor. As a result, each exam included 50 three-option questions (two distractors) and 50 four-option questions (three distractors). All items were weighted equally in the final score, with error penalties adjusted according to the number of distractors: –0.5 points per error in three-option questions and –0.33 points per error in four-option questions, in line with prior recommendations to ensure fairness in scoring [4].

The following variables were collected for each item:

- Difficulty index [9]: proportion of students who answered the item correctly.
- Point-biserial coefficient of the right answer [9]: correlation between selecting the correct option or not and the total score on the exam.
- Distractor efficiency: number of low-functioning distractors—selected by less than 5% of students [1].
- Question type: classified as either factual or clinical case-based by the first author.
- Technical quality was assessed using the 19-item defect checklist described by Tarrant et al. [2], which comprises predefined and operationalized criteria for common item-writing flaws. For analytical purposes, items were coded as a categorical variable (no defects versus at least one defect). The review was conducted by the first author. Given the structured nature of the checklist and its explicit criteria, the assessment was considered largely objective. Ambiguous cases were discussed and resolved in consultation with senior authors with formal training in medical education and prior experience in item analysis to enhance consistency.

Items were categorized according to their difficulty index (easy > 0.7 versus non-easy ≤ 0.7) and discriminative power (highly discriminative point-biserial > 0.3 versus not-highly discriminative point-biserial ≤ 0.3) [9]. Additionally, we applied a composite indicator of item quality to identify high-quality MCQs. This indicator combines a difficulty index within the 0.45–0.75 range and a point-biserial > 0.20, identifying questions that are of moderate difficulty and moderate or high discriminative value. While alternative thresholds may also be defensible depending on context, the criteria adopted in this study are grounded in established psychometric literature in medical education, which consistently recommends that items within an intermediate difficulty range and demonstrating acceptable discrimination provide optimal differentiation between higher- and lower-performing students [9]. By integrating both difficulty and discrimination parameters, the composite indicator provides a more comprehensive framework for evaluating the performance of test items. In addition, such analysis ensures that examinations more accurately differentiate between higher- and lower-performing students, thereby enhancing their validity as measures of learning outcomes. From this point forward, items meeting these criteria will be referred to as preferred questions, emphasizing their suitability as exemplary items for high-quality assessment design.

We also recorded whether more than 10 % of the students left the item unanswered.

### Statistical Analysis

Continuous variables were reported as mean ± standard deviation when normally distributed and as median with interquartile range when distributional assumptions were not met. Categorical variables were expressed as absolute counts and percentages.

Group comparisons between two- and three-distractor formats were performed using Student’s t-test or the Mann–Whitney U test for continuous variables, and chi-squared tests for categorical variables, as appropriate.

Univariate analyses were conducted separately for each predefined outcome variable: [1] item difficulty (categorized as easy, DI > 0.70 vs. non-easy, DI ≤ 0.70), [2] high discriminative capacity (point-biserial > 0.30 vs. ≤ 0.30), and [3] preferred question status (composite indicator). The independent variables examined in univariate analyses included number of distractors (two vs. three), question type (factual vs. clinical case-based), presence of item-writing flaws (none vs. ≥1), and presence of low-functioning distractors (none vs. ≥1).

Variables demonstrating a p-value < 0.20 in univariate analyses for each specific outcome were entered into separate multivariable logistic regression models to identify factors independently associated with that outcome. Adjusted odds ratios (ORs) with 95% confidence intervals were calculated. Statistical significance was defined as p < 0.05.

All analyses were performed using IBM SPSS Statistics version 20 (IBM Corp., Armonk, NY, USA).

### Ethical Considerations

The Research Ethics Committee of the University of Navarra and the governing board of the School of Medicine approved the study protocol (Approval No. 2022.143).

## Results

### General characteristics

A total of 198 MCQs were analyzed (Table 1), evenly distributed between items with two distractors (n = 98) and three distractors (n = 100). Two items were excluded due to major writing flaws. The data set was obtained from the participation of 211 and 212 second-year medical students in the System and Organ Pathology I and II courses, respectively.

**Table 1:** Overall characteristics and comparison according to distractor format (n = 198).


VARIABLE	OVERALL (n = 198)	2 DISTRACTORS (n = 98)	3 DISTRACTORS (n = 100)	p-VALUE

**Nature of question**				0.383

Factual, n (%)	89 (44.9)	41 (41.8)	48 (48)	

Clinical case, n (%)	109 (55.1)	57 (58.2)	52 (52)	

**Low-functioning distractors (LFD)**				

0 LFD, n (%)	56 (28.3)	38 (38.8)	18 (18)	0.001

≥1 LFD, n (%)	142 (71.7)	60 (61.2)	82 (82)	

**Left unanswered >10%, n (%)**	113 (57)	62 (63.3)	51 (51)	0.067

**Technical flaws**				0.045

No flaws, n (%)	105 (53)	59 (60.2)	46 (46)	

With flaws, n (%)	93 (47)	39 (39.8)	54 (54)	

Difficulty index*	0.68 (0.48–0.84)	0.68 (0.35)	0.68 (0.37)	0.931

Point-biserial**	0.28 (0.12)	0.29 (0.16)	0.29 (0.15)	0.708

Preferred questions***, n (%)	72 (36.4)	38 (38.8)	34 (34)	0.485


*Median (interquartile range). **Mean (standard deviation). ***Difficulty index between 0.45 and 0.75, and a point-biserial > 0.20.

A total of 28.3% of the items included no low-functioning distractors, and 57% were left unanswered by over 10% of students.

Of the total items, 55% were structured as clinical vignettes, while 45% were factual questions. Regarding technical quality, 53% of the questions were free of item-writing flaws according to the criteria described by Tarrant et al. The remaining 93 items (47%) exhibited at least one defect, the most frequent being: absence of reference to overly specific content (22.6%), inclusion of unnecessary or irrelevant information (25.8%), and use of negative phrasing in the question stem (48.4%).

In terms of psychometric performance, the median item difficulty index was 0.68 (interquartile range: 0.48–0.84) and the mean point-biserial correlation was 0.28 (SD: 0.12). Finally, 36.4% of items met the predefined composite endpoint for *preferred questions*, defined as a difficulty index between 0.45 and 0.75 and a point-biserial > 0.20.

### Analysis of item characteristics based on the number of distractors

No statistically significant differences were observed between items with two versus three distractors in terms of question type (factual versus clinical case-based), difficulty index, or discriminatory power, as measured by the point-biserial coefficient (Table 1).

Items with two distractors demonstrated a lower frequency of item-writing flaws (39.8% versus 54%; p = 0.045), and a higher proportion of questions without low-functioning distractors (38.8% versus 18%, p = 0.01). Specifically, among the two-distractor items, 39.8% contained one low-functioning distractor and 21.4% contained two. In the three-distractor group, 31% of items included one low-functioning distractor, 33% included two, and 18% included three.

Based on the composite criterion for high-quality items, a total of 72 questions (36.4%) met the quality threshold, and were therefore defined as *preferred questions*, with no statistically significant differences observed between the two- and three-distractor groups (38.8% versus 34%, respectively).

We observed a higher proportion of items with only two distractors being left unanswered by more than 10% of students; however, this difference did not reach statistical significance (p = 0.067).

### Analysis of factors potentially associated with item difficulty index (DI)

In the comparative analysis (Table 2) between items classified as easy (DI > 0.7) and non-easy (DI ≤ 0.7), a statistically significant difference was observed in the proportion of factual questions: 56 out of 95 (59%) of the easier items were factual, compared to 33 out of 103 (32%) in the non-easy group.

**Table 2:** Analysis of factors potentially associated with item difficulty (ID).


VARIABLE	EASY ITEMS DI > 0.7 (n = 95)	NON-EASY ITEMS DI ≤ 0.7 (n = 103)	UNIV. p-VALUE	ADJUSTED OR (95% CI)	ADJ. p-VALUE

**Number of distractors**			0.780	—	—

2 distractors, n (%)	48 (50.5%)	50 (48.5%)			

3 distractors, n (%)	47 (49.5%)	53 (51.5%)			

**Nature of the question**			<0.001		

Factual, n (%)	56 (58.9%)	33 (32.1%)		2.62 (1.38–4.99)	0.003

Clinical case, n (%)	39 (41.1%)	70 (67.9%)		Reference	

**Technical flaws**			0.109		

No flaws, n (%)	56 (58.9%)	49 (47.6%)		Reference	

With flaws, n (%)	39 (41.1%)	54 (52.4%)		1.34 (0.71–2.56)	0.369

**Low-functioning distractors (LFD)**			<0.001		

≥ 1 LFD, n (%)	88 (92.6%)	54 (52.4%)		Reference	

0 LFD, n (%)	7 (7.4%)	49 (47.6%)		0.10 (0.04–0.24)	<0.001

**Point-biserial (>0.30)**			0.212	—	—

> 0.30, n (%)	49 (51.6%)	44 (42.7%)			

≤ 0.30, n (%)	46 (48.4%)	59 (57.3%)			


A statistically significant association was also found between the presence of low-functioning distractors and item ease. Specifically, 92.6% of the easy items contained at least one low-functioning distractor, compared to 52.4% of the non-easy items (p < 0.001).

No statistically significant associations were observed between item difficulty and the number of distractors, the presence of technical flaws, or point-biserial coefficient.

In multivariate regression analysis (Table 2), the nature of the question emerged as an independent predictor of item difficulty. Factual questions were significantly more likely to be classified as easy compared to clinical case-based questions (OR: 2.62; 95% CI: 1.38–4.99; p = 0.003). Conversely, the absence of low-functioning distractors was inversely associated with item ease (OR: 0.1; 95% CI: 0.04–0.24; p < 0.001).

### Analysis of factors potentially associated with high discriminative power

No statistically significant differences were observed in the proportion of items with point-biserial > 0.30 according to the number of distractors, question nature, presence or absence of item-writing flaws, or presence of low-functioning distractors.

Although a higher proportion of easy items (DI > 0.7) was observed among those with point-biserial > 0.30 (52.7% versus 47.3%), this difference did not reach statistical significance (p = 0.212).

### Analysis of factors potentially associated with the composite high-quality item criterion (Preferred Questions)

Table 3 presents the analysis of factors associated with questions classified as high quality (*preferred questions*), defined as those with a difficulty index between 0.45 and 0.75 and a point-biserial coefficient > 0.20. No statistically significant differences were found in the proportion of high-quality questions according to the number of distractors or question nature (factual versus clinical case-based).

**Table 3:** Analysis of factors potentially associated with *preferred questions*.


VARIABLE	COMPARISON	% PREFERRED QUESTIONS IN EACH CATEGORY	p (CHI^2^/FISHER)	ODDS RATIO (95% CI)	p (REGRESSION)

**Presence of technical flaws**	No flaws vs. ≥1 flaws	31/105 (29.5%) vs. 41/93 (44.1%)	0.034	0.57 (0.31–1.03)	0.061

**Low-functioning distractors**	0 LFD vs. ≥1 LFD	27/56 (48.2%) vs. 45/142 (31.7%)	0.029	1.87 (0.99–3.56)	0.055

**Number of distractors**	2 vs. 3 distractors	38/98 (38.8%) vs. 34/100 (34%)	0.485	Not included	—

**Nature of the question**	Clinical case vs. Factual	32/89 (35.9%) vs. 40/109 (36.7%)	0.914	Not included	—


Preferred questions: questions with difficulty index between 0.45 and 0.75 and point-biserial > 0.20. Binary logistic regression was performed using the Enter method. Variables with p < 0.20 in the univariate analysis were included in the model.

The proportion of items free from item-writing flaws was significantly lower in the high-quality group compared to the lower-quality group (31/72, 43.1% versus 74/126, 58.5%; p = 0.034). Finally, the absence of low-functioning distractors was significantly associated with overall item quality: 27/72 (37.5%) of *preferred questions* had no low-functioning distractors, compared to 29/126 (23%) of non-preferred questions items (p = 0.029).

In multivariate analysis (Table 3), none of the included variables emerged as independent predictors, although both showed a trend toward statistical significance. The absence of low-functioning distractors was associated with a higher likelihood of being classified as a high-quality question (OR: 1.87; 95% CI: 0.99–3.56; p = 0.055), whereas the absence of item-writing flaws showed an inverse, non-significant association (OR: 0.57; 95% CI: 0.31–1.03; p = 0.061).

## Discussion

The findings of this study provide further and consistent evidence regarding the impact of reducing the number of distractors from 3 to 2 in MCQs within undergraduate medical education assessment. In line with previous research [3,4,10], our results demonstrate that reducing the number of distractors from three to two does not compromise item difficulty or discriminative power, as measured by the difficulty index and the point-biserial coefficient, respectively. This finding aligns with Rodríguez’s systematic review [3], which concluded that three-option MCQs are psychometrically optimal, and with more recent studies showing that items with fewer distractors may even perform better [11,12].

In our study, items with two distractors contained significantly fewer low-functioning distractors, a factor known to enhance item quality and difficulty. Conversely, three-distractor items were more likely to include low-functioning distractors, which may facilitate test-taking strategies such as elimination. This pattern effectively reduces the number of plausible options to two, while still applying a lower penalty for incorrect responses compared to items originally written with two distractors. This phenomenon may help explain why items with two distractors in our study were more frequently left unanswered by more than 10% of students.

Importantly, the number of distractors did not emerge as an independent predictor of global item quality, which we defined using a composite criterion that included a moderate difficulty index and a moderate or high discriminative coefficient.

A key strength of our study lies in its randomized design, which allowed for the direct comparison of matched questions differing only in the number of distractors, thus minimizing confounding and selection bias. The absence of significant differences in discrimination index, point-biserial coefficient, and attainment of high-quality status between the two- and three-distractor formats reinforces the hypothesis that distractor quality, not quantity, is the main determinant of item performance. This principle has been consistently emphasized in the literature, particularly by Haladyna et al. [4], who stressed the importance of developing plausible distractors that reflect common student misconceptions.

In our study, items with two distractors showed fewer item-writing flaws and fewer low-functioning distractors, which may reflect the reduced cognitive burden involved in generating plausible distractors. From a practical standpoint, this finding suggests that reducing the number of distractors increases the likelihood that all included options serve a real role in discriminating student knowledge. This conclusion aligns with previous literature highlighting the inherent difficulty of constructing technically valid distractors [6] and studies showing that the inclusion of ineffective distractors can introduce unnecessary confusion and undermine item validity [1]. Moreover, eliminating low-quality distractors may help reduce cognitive fatigue for students and focus their attention on conceptually relevant aspects of the item.

Additionally, our analysis of factors associated with item difficulty and discrimination revealed that factual questions tend to be easier, whereas clinical case-based questions are more complex and better at differentiating between levels of student performance. In our dataset, factual questions were more likely to include low-functioning distractors, suggesting that designing high-quality distractors for factual content may be more challenging. These findings are consistent with current trends in medical education that emphasize the assessment of clinical competence and applied reasoning, rather than mere recall. Clinical contextualization enhances alignment between assessment and the learning objectives [7]. Moreover, clinical scenarios may better prepare students for real-world practice by reinforcing the theory-to-practice connection.

Our study also found that the presence of low-functioning distractors was associated significantly with easier items. Poorly constructed distractors, once recognized and discarded, increase the likelihood of selecting the correct answer. This fact reinforces the importance of critically reviewing distractor functionality during post-exam item analysis, as suggested by Fozzard et al. [5] and Chauhan et al. [13], particularly in high-stakes assessments.

Interestingly, our data showed a paradoxical trend: the absence of item-writing flaws was inversely associated—though not significantly—with inclusion in the *Preferred Questions* group. If confirmed, this finding may underscore the greater impact of distractor content and functionality on item performance, relative to formal writing quality alone.

Our findings are consistent with those of Sridharan and Sivaramakrishnan (2025), who reported no significant differences in discrimination indices across option formats and a lower risk of non-functioning distractors in three-option MCQs. Notably, their analysis also highlighted shorter response times and higher difficulty indices in reduced-option formats. Although our study did not evaluate response time, the convergence of findings supports the notion that distractor quality, rather than quantity, is the primary determinant of item performance [15].

Taken together, these findings support a progressive transition toward the use of carefully constructed two-distractor items. This strategy could enhance exam development efficiency, reduce writing errors, and maintain appropriate levels of item discrimination. This approach not only optimizes faculty workload but also aligns with robust psychometric principles and current recommendations in medical education [4,14,15].

## Limitations

This study has several limitations that should be considered when interpreting its findings. First, although the randomized design allowed partial control over the allocation of items with different numbers of distractors, the statistical significance of the results may be affected by the fact that two-distractor items were originally written with three distractors. One distractor was deliberately removed by the faculty member responsible for each item. This process may have compromised item quality and introduced bias related to the judgment of the individual author, despite the use of standardized instructions to guide the removal process.

Second, the evaluator assessing item-writing flaws was a member of the research team and aware of the study aims, and no independent, second, blinded reviewer was involved, which could have introduced additional bias. To mitigate this situation, a structured checklist [2], based on predefined and operationalized criteria, was used to guide the assessment.

In addition, items were authored by different instructors, which may have introduced variability in writing quality and style across the dataset.

Third, the analysis was limited to two second-year courses at a single institution, which restricts the generalizability of the results to other curricular contexts or academic levels.

Finally, the study did not assess the impact of question format on individual student performance or students’ qualitative perceptions of the examination—both important aspects in the context of formative, learner-centered assessment. Future studies should address these aspects to provide a more comprehensive understanding of item performance and student experience.

## Conclusion

The findings of this study support the validity of using multiple-choice questions with two distractors as an effective and technically sound alternative to the traditional three-distractor format. This structure was associated with a lower rate of item-writing flaws and greater distractor functionality, without negatively affecting item difficulty or discriminative power.

Taken together, these results have practical implications for the design of assessment tools in medical education. Reducing the number of distractors not only optimizes faculty workload but also contributes to improving the overall quality of examinations.

## Data Availability

The dataset analyzed in the current study is available from the corresponding author on reasonable request.
